# Insights into Acute Pancreatitis: Pathogenesis, Diagnosis, and Management

**DOI:** 10.3390/jcm15082819

**Published:** 2026-04-08

**Authors:** Silvia Carrara, Federico Cassano, Maria Terrin, Marco Spadaccini

**Affiliations:** 1 Department of Biomedical Sciences, Humanitas University, Pieve Emanuele, 20072 Milan, Italy; 2 Department of Gastroenterology, IRCCS Humanitas Research Hospital, Rozzano, 20089 Milan, Italy

**Keywords:** acute pancreatitis, etiology, fluid resuscitation, artificial intelligence, endoscopy

## Abstract

This narrative review integrates landmark studies, recent publications, and major clinical guidelines to highlight the current state of the art concerning acute pancreatitis, a well-known yet still challenging condition. We will focus on recent practice transitions and future perspectives arising from advances in diagnostic imaging and interventional endoscopy. **Pathogenesis and etiology**: We carry out an overview of the fundamental mechanisms underlying acute pancreatitis, followed by an analysis of both common and uncommon causes, along with emerging evidence regarding idiopathic forms. **Diagnosis and risk stratification**: We pursue two objectives: on one hand, to emphasize the enduring importance of clinical assessment in the diagnosis of acute pancreatitis; on the other, to analyze the increasingly central role that imaging has acquired over recent decades. Identification of patients at higher risk for complications or an unfavorable prognosis is crucial. Several scoring systems have been proposed over the past decades, but with limited impact on daily clinical practice. **Treatment**: Therapeutic approaches have undergone significant revisions over time. Our objective is to provide an overview of the current standards together with best evidence-based medical approaches, targeted and interventional therapies, with focus on the endoscopic ones. Furthermore, we want to clarify the importance of nutrition and its proper management. **Conclusions**: Acute pancreatitis continues to stimulate discoveries and improvements in clinical management. We will place emphasis on unmet needs and emerging innovations that may importantly influence future practice also promoting evidenced-based standards of care.

## 1. Introduction

Acute pancreatitis (AP) is one of the most common gastroenterological disorders worldwide with an incidence of 33–74 cases per 100,000 person years [1]. This leads to approximately 280,000 annual hospital admissions and costs exceeding 2.5 billion dollars in the United States [2]. Global incidence has been reported to have increased by 3%, with large regional differences: North America and Europe have experienced the highest increases in incidence, 3.67% and 2.6–2.77%, respectively, whereas incidence has remained stable in Asia [3]. The European trend is confirmed in a recent study with a reported incidence increment of 2.6% in England [4]. Most of the patients with AP present with a mild acute form which is self-limiting; however, there is a portion of patients, approximately 20%, who develop moderate-to-severe pancreatitis with necrosis or organ failure, carrying a mortality rate between 20 and 40% [5]. This review aims to provide a clinically oriented and critical synthesis of current evidence on AP, focusing on areas of evolving practice and controversy. A non-systematic literature search was conducted in PubMed and Embase for relevant publications from January 2016 to January 2026. Priority was given to major international guidelines and to high-quality clinical studies, including randomized controlled trials and meta-analyses published shortly before or after guideline release. The search strategy combined the following keywords: “acute pancreatitis”, “etiology”, “risk stratification”, “management”, “fluid resuscitation”, and “fluid therapy”, combined when appropriate with additional terms such as “nutrition”, “enteral nutrition”, “endoscopic intervention”, “guidelines”, “complications”, and “artificial intelligence”. Earlier relevant studies were identified through manual screening of reference lists, when necessary to contextualize current clinical practice. Study selection was performed independently by two authors based on their relevance to the clinical management and their contribution to the understanding of current diagnostic and therapeutic strategies. Studies were excluded if they focused on pediatric populations, were not available in full text, or were deemed not relevant after full-text evaluation. The final selection of studies was based on the authors’ assessment of methodological quality, study design, and clinical relevance.

## 2. Pathogenesis and Etiology

AP is primarily caused by damage to the pancreatic acinar cells, triggering the release and activation of trypsinogen into trypsin within the acini. Trypsin then activates other digestive enzymes, the kinin system, and the complement cascade, leading to autodigestion of the pancreatic parenchyma [6].

Biliary disease and alcohol remain the leading etiologies, although with marked geographical variability [6,7]. Biliary etiology accounts for 40–60% of cases worldwide [7,8,9] ranging from 50% of cases in Europe and increasing up to 78% in Latin America, but representing less than 28% of cases in India, where alcohol-induced AP is prevalent [7]. Alcohol-induced AP has a global incidence of around 21–35%, up to 44% of all cases in India [7,9].

Etiologic distribution also affects demographic trends of the disease, which generally affects males and females equally worldwide, particularly in Europe and North America. In regions with a high incidence of biliary etiology, such as Latin America, the majority of patients are female (72%). Conversely, in India, due to a high incidence of alcoholic etiology, the trend is reversed, with the majority of patients being male (74%) [7].

**Figure 1:**
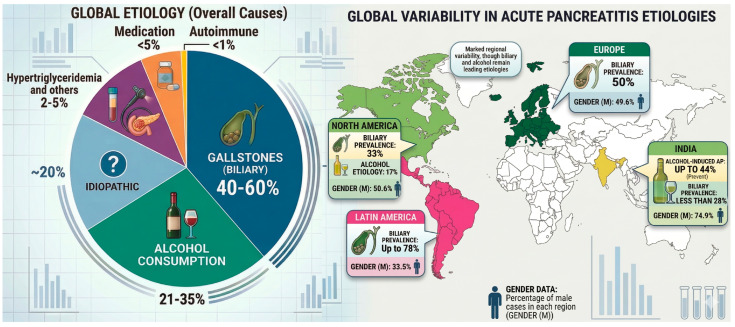
Main etiologies with their geographical distribution.

Less common causes include other toxic and obstructive factors, such as hypertriglyceridemia, smoking, hypercalcemia, and certain medications, as well as genetic, autoimmune, and idiopathic forms [10]. Hypertriglyceridemia accounts for 2–5% of all causes of AP [11]. Drug-induced AP causes fewer than 5% of the cases [8], while autoimmune pancreatitis is even less common, representing less than 1% of AP cases [11]. In around 20% of cases, the cause cannot be established, and it is defined as idiopathic pancreatitis [9]. The proportion of patients with idiopathic AP increases with age [11], highlighting the need for thorough diagnostic work-up to prevent further recurrences. Figure 1 reports the major etiologies with their geographical distribution.

Pancreatic duct pressure might play a crucial role in the pathologic process: published studies have demonstrated that an increase in duct pressure, for instance, due to an obstruction as in a biliary etiology or cancer, can result in enhanced ductal permeability leading to the subsequent spread of pancreatic enzymes [12]. A similar mechanism is shared by other etiologies that can cause a transient obstruction, mainly post-ERCP AP, and, although data supporting these conditions are controversial, pancreas divisum and sphincter of Oddi dysfunction [11]. Lastly, localized masses or mucus obstructing the ductal system should also be considered, in particular when an IPMN (Intraductal Papillary Mucinous Neoplasm) or a pancreatic duct adenocarcinoma are present [10]. Thus, occult malignancy should be particularly suspected in patients over 40 years of age, especially in the context of an unclear etiology or a recurrent course, and in the presence of other risk factors for pancreatic cancer [8,9].

The pathogenesis of alcohol-induced AP remains mostly unknown [6]. Although it is well-established that the risk of developing pancreatitis increases with alcohol consumption, only a minority of heavy drinkers experience clinically evident pancreatitis, suggesting that additional factors may contribute to the development of AP [13]. According to some studies, alcohol could lower the threshold for trypsin activation within the pancreas, causing cellular necrosis [14], partly because it is metabolized by pancreatic and stellate cells, producing oxidative and non-oxidative metabolites [13]. Other authors argue that ethanol has been shown to increase sphincter of Oddi tone and this mechanism, together with the ethanol-induced rise in pancreatic secretion, might lead to elevated ductal pressure [12]. It is therefore plausible that alcohol could potentiate pancreatic injury from other underlying environmental (e.g., smoking) and genetic risk factors [8].

The mechanism underlying hypertriglyceridemia-induced AP remains a subject of debate. One theory suggests that elevated triglyceride levels are hydrolyzed by pancreatic lipase within the gland, resulting in the production of high levels of free fatty acids that subsequently form micellar structures damaging pancreatic acinar cells and vessels. Alternatively, vascular hyperviscosity, due to high levels of chylomicrons, may lead to impaired blood flow, resulting in ischemia and inflammation [15]. Traditionally, serum triglyceride levels >1000 mg/dL are considered the threshold to attribute AP to hypertriglyceridemia [9]; however, a recent study suggests a potential lower threshold, emphasizing the significance of values exceeding 177 mg/dL, with a concentration-dependent increase in risk, reaching a hazard ratio (HR) of 8.7 at levels ≥ 443 mg/dL [15]. Also, findings from a recent multicenter Italian cohort study suggest that moderate-to-severe hypertriglyceridemia should not be regarded as an incidental finding, as it may contribute to pancreatic injury [16].

Inappropriate release of intracellular calcium, enhanced entry of extracellular calcium, or defective extrusion mechanisms result in an increase in calcium levels in the cytoplasm. High cytoplasmic calcium levels can lead to the premature activation of trypsinogen, resulting in acinar injury [6]. Approximately 90% of cases of hypercalcemia are attributed to primary hyperparathyroidism or hypercalcemia associated with malignancies. A smaller percentage is caused by genetic disorders, sarcoidosis, and chronic kidney disease. Serum calcium levels ≥12.0 mg/dL are considered indicative of hypercalcemic pancreatitis [10].

More than 500 drugs are reported in the WHO database as potentially causing AP. However, most drug reports include only case reports and even rechallenges could be deceptive due to the high proportion of patients with AP recurrence even without any medication. Only three drugs have been shown to be associated with acute pancreatitis based on evidence from randomized controlled clinical trials (RCTs): didanosine, azathioprine, and 6-mercaptopurine [17]. Of note, although GLP1 receptor agonists were associated with AP in case–control and pharmacoepidemiology studies [17], recent large observational studies and a meta-analysis suggest no significant increase in risk, and current evidence does not support routine discontinuation in patients without prior pancreatitis [18,19,20].

Among less common etiologies, genetic causes should be taken into account, in particular mutations in PRSS1, SPINK1 and CFTR genes [8]. CFTR variants have been reported in up to 12% of the patients with pancreatic disorders [21]. However, the clinical relevance of these variants remains debated, especially for SPINK1 and, even more, for heterozygous CFTR mutations, which may act as predisposing factors rather than being directly causative. Infections are a rare cause, generally sustained by a viral agent such as EBV, CMV, or mumps, but parasites and nematodes could also be found [8].

## 3. Diagnosis and Risk Stratification

In accordance with the revised Atlanta classification, the diagnosis of AP is established if at least two out of the three following criteria are met: (1) abdominal pain consistent with AP, (2) serum lipase or amylase levels that are at least three times higher than the upper limit, and (3) imaging findings suggestive of AP on computed tomography (CT), magnetic resonance imaging (MRI), or transabdominal ultrasound (US) [22]. Since its publication, this classification has been widely adopted to these days [9,23]. Abdominal pain represents the hallmark presenting symptom: typically persistent, with radiation to the back, and it is sometimes exacerbated by food intake. Nausea, vomiting, and low or moderate fever are other common symptoms [6]. Regarding laboratory evaluation, both amylase and lipase are valuable markers; however, lipase has a longer half-life and higher specificity, resulting in a wider diagnostic window than amylase [24], and could be preferred in patients who present later after pain onset [9]. It should also be recognized that elevated amylase or lipase levels are not specific for pancreatic injury, as a variety of extra-pancreatic conditions may produce similar biochemical abnormalities, including peritonitis, appendicitis, renal or gynecological diseases, parotitis, and macroamylasemia [8]. Moreover, there is no correlation between the levels of amylase and lipase and the clinical severity of the disease, and serial monitoring does not provide meaningful prognostic information [25].

Regarding imaging, current international guidelines suggest that MRI, CT, and abdominal ultrasound are viable radiological techniques for diagnosing pancreatic inflammation. Nevertheless, in this specific context, contrast-enhanced computed tomography (CECT) remains the most commonly employed imaging modality in clinical practice [23]. MRI has been reported to offer higher sensitivity and specificity compared to CECT while also allowing for a more detailed evaluation of the pancreatic parenchyma and the common bile duct (CBD), particularly when implemented with cholangiopancreatography (MRCP). However, MRI requires longer acquisition times, local expertise, and higher costs; therefore, CECT remains the preferred technique in many clinical settings [26].

As the diagnosis of AP can often be established solely through clinical and laboratory tests, in such cases, imaging should be performed after 48–72 h (and up to 96 h according to last international guidelines) to detect any complications or clarify the underlying cause of AP [9,23]. Identifying the etiology requires a comprehensive patient history, including previous episodes of AP, gallstone disease, alcohol consumption, medication use, known hypertriglyceridemia, trauma, and recent ERCP. Additionally, laboratory tests for liver enzymes, calcium levels, and triglycerides should be conducted at presentation [23]. Liver enzymes are particularly useful in identifying a biliary cause. Increasing ALT levels correlate with higher specificity and positive predictive value for gallstone pancreatitis, and serum levels exceeding 150 IU/L within the first 48 h have demonstrated a positive predictive value exceeding 85% [27,28]. US remains central in the evaluation of suspected biliary etiology by detecting gallstones or a dilated CBD [9,23], although it could also identify complications such as peripancreatic fluid collections [26]. The combined assessment of liver enzymes and CBD diameter on US enables the stratification of patients into three groups based on their pre-test probability of CBD gallstones: low likelihood, absence of both enzyme elevation and CBD dilation; intermediate likelihood, marked by enzyme elevation or CBD dilation; and high likelihood, where both alterations are found [29].

Clinical and radiological assessments are not only pertinent to the diagnostic process but also play a key role in the risk stratification of AP. Although most patients experience mild or moderate AP, approximately 20% of patients progress to a more severe form of the disease, and clinical deterioration may occur even after an initially mild presentation [8]. Radiologically, the Revised Atlanta Classification distinguishes two major forms of AP: interstitial edematous pancreatitis and necrotizing pancreatitis. The first one is characterized by a diffuse or localized enlargement of the gland due to inflammatory edema and typically carries a more favorable prognosis. Necrotizing pancreatitis, which develops in approximately 10% of patients, on the other hand, involves necrosis of the pancreatic parenchyma, peripancreatic tissue, or both, the latter being the most prevalent form. As early imaging may lead to underestimation of its extension, it is not recommended [22]. Necrotizing disease is associated with a poorer prognosis, with a mortality rate that rises from 15% up to 30–39% in case of infection of the necrotic tissue [30]. Due to the potential for necrosis’ underestimation, artificial intelligence (AI)-based diagnostic models are currently being studied as tools to assist clinicians and radiologists in diagnosing AP from CT scans, with a reported area under the curve (AUC) of 0.85 while also being capable of discriminating between different segmental lesions [31].

Local complications include pancreatic and peripancreatic collections, gastric outlet dysfunction, portal or splenic vein thrombosis, and colonic necrosis. Pancreatic and peripancreatic collections are further classified according to timing, imaging characteristics, and the underlying type of pancreatitis. Acute peripancreatic fluid collections occur within the first four weeks of interstitial edematous pancreatitis and consist solely of fluid without associated necrosis. After encapsulation, which typically occurs beyond four weeks, the collections are termed pancreatic pseudocysts. In necrotizing pancreatitis, an acute necrotic collection may develop, containing both fluid and necrotic debris, and may later evolve into walled-off necrosis (WON), an encapsulated collection of pancreatic and/or peripancreatic necrotic tissue [22].

From a clinical perspective, the Revised Atlanta Classification defines mild AP as the absence of both organ system failure and local complications, whereas persistent organ failure lasting more than 48 h is classified as severe [22]. However, risk stratification remains a key unmet need in the management of AP. Traditional scoring systems include the Acute Physiology and Chronic Health Evaluation II (APACHE II), the Bedside Index of Severity in AP (BISAP), Ranson’s score, the Computed Tomography Severity Index (CTSI) and the Systemic Inflammatory Response Syndrome (SIRS). A comparative study shows good performance in predicting severity and mortality for all scores, with an AUC of 0.81 and 0.82 for BISAP, 0.94 and 0.95 for Ranson’s, 0.78 and 0.94 for APACHE II, and 0.84 and 0.83 for CTSI, respectively [32]. A meta-analysis including more than 30 studies shows comparable AUCs for predicting AP severity with non-significant differences between the scores; however, regarding mortality, it demonstrates a higher predictive value for APACHE II, with an AUC of 0.91 compared to 0.79 for CTSI, 0.87 for BISAP, and 0.87 for Ranson’s [33]. On the other hand, it has been suggested that expert clinician judgment and the simple SIRS score are as effective as any complex scoring system [11]. In particular, SIRS on day one has been reported to have high negative predictive values (98–100%), but very low positive predictive values (6–17%) [34]. Overall, the scores showed broadly comparable predictive performance but are limited by complexity, delayed applicability (given the necessity in some scores to wait 48 h), or suboptimal integration into routine clinical practice. Consequently, simpler and dynamic tools such as the SIRS criteria and bedside clinical assessment continue to be widely used. The international guidelines recommend using SIRS criteria at admission and at 48 h for predicting severity, along with C-reactive protein (CRP) and Interleukin-6 (IL-6) levels, whereas the American College of Gastroenterology (ACG) Guidelines suggest using SIRS criteria as well but do not recommend using CRP because of its latency. Instead, they suggest using blood urea nitrogen (BUN) and hematocrit (HCT) to assess any sign of hypovolemia [9,23]. There are also many known risk factors for a poor prognosis, such as age [35], severe comorbidities, BMI, and hypertriglyceridemia that should be considered when evaluating risk stratification [23].

Several AI models have been proposed to stratify severity upon admission and the necessity for intensive care unit (ICU) admission, demonstrating strong predictive capabilities, but with some differences between the models’ performances [36,37]. Xgboost, one of the most commonly used models, demonstrated an AUC of 0.95 and 0.87 for predicting access to ICU in internal and external validation cohorts, respectively [38]. The rising accessibility of large language models (LLMs) is generating growing interest in their potential use of these tools. A study conducted using Gemini revealed an accuracy of 85% and 82% in identifying mild and severe cases of AP, respectively, combined with the ability to compute APACHE II and Ranson’s scores with comparable precision to medical professionals [39]. LLMs are also being evaluated for their role in disease management. Gemini recorded an overall guideline-adherence rate of 82.4%, although the model’s suggestions were noted to be conservative, with a tendency to delay enteral nutrition and an over-prescription of antibiotic prophylaxis; in contrast, ChatGPT 3.5 recorded a lower accuracy rate of 71%. Overall LLMs may represent a promising clinical decision-support tools, especially in hospitals with limited experience; however, further validation and clinical supervision are essential before routine implementation [39,40].

## 4. Treatment

### 4.1. General Treatment

There are three fundamental pillars of treatment: fluid resuscitation, pain management, and nutritional support. Recent evidence has resulted in a transition in practice toward moderate, goal-directed fluid resuscitation, and overly aggressive hydration is now discouraged due to the increased risk of fluid overload without clinical benefit. In this setting, Lactated Ringer’s (LR) solution is preferred over normal saline (NS) based on its anti-inflammatory and metabolic advantages. Furthermore, nutritional management has significantly evolved, with growing evidence supporting early oral feeding over a nil per os strategy, thereby challenging the traditional “gut rest” concept.

Inflammation-induced endothelial dysfunction, characterized by capillary leak syndrome, leads to a loss of intravascular volume, resulting in hypoperfusion of organs and accumulation of interstitial fluid [41]. The primary objective of fluid resuscitation is to rectify regional hypoperfusion of the pancreas, a condition that has been demonstrated in animal models to be associated with necrosis [42]. Based on this pathophysiological rationale, previous guidelines suggested aggressive hydration with 5–10 mL/kg/h of fluid therapy until resuscitation goals are reached, including a heart rate <120 bpm, mean arterial pressure between 65 and 85 mmHg, urinary output >0.5–1 mL/Kg/h and hematocrit between 35 and 44% [27,43]. Targeted therapy is also recommended in the American Gastroenterological Association (AGA) guidelines, which are more recent than those previously mentioned, without precise quantification of volumes and rates, but cautioning against overly aggressive fluid therapy [44]. However, recent clinical evidence has raised concerns regarding the safety of such aggressive strategies. In particular, the WATERFALL trial demonstrated that moderate fluid resuscitation significantly reduced the risk of fluid overload while achieving comparable efficacy in preventing disease progression compared to aggressive fluid resuscitation [42]. More recent guidelines reflect this evolving perspective. The 2024 ACG guidelines recommend a moderately aggressive fluid resuscitation strategy, along with frequent reassessment of the patient’s response supported by the evaluation of BUN levels [9]. Similarly, the 2025 International guidelines suggest a moderate infusion rate of approximately 1.5 mL/kg/h, with the option of an initial fluid bolus in patients presenting with hypovolemia or hypotension [23]. Several meta-analyses have attempted to clarify the optimal fluid resuscitation strategy, although their findings remain partially heterogeneous. The meta-analysis by Kumari et al. compared low, moderate, and aggressive fluid resuscitation including both RCTs and observational studies. Moderate hydration was associated with improved clinical outcomes compared to low-volume resuscitation, including lower persistence of SIRS, reduced organ failure, and decreased mortality. Similar results favoring high fluid volumes were also observed when compared to low or moderate hydration strategies [45]. In contrast, RCT-based meta-analyses by Ehsan et al. and Evan et al., respectively, reported an increased mortality with early aggressive fluid resuscitation, and higher rates of sepsis and longer hospital stays with aggressive hydration, without significant differences in other clinical outcomes [46,47]. Furthermore, subgroup analyses suggested that the risk associated with aggressive hydration may increase with disease severity [47]. The variability between the studies may be attributed to several methodological issues that were inherent in the studies, such as the small number of patients in the RCTs, variability in fluid resuscitation protocols, and the solution used for resuscitation. Additionally, variability in the studies has been observed in the patient population and the severity of AP. In clinical practice, substantial regional variability in fluid resuscitation strategies has been reported: European hospitals tend to have a lower fluid infusion volume in the first 24 h (2.5 L) compared to Indian and North and Latin American ones (3–3.2 L) [7]. Overall, the available evidence appears to support a moderate resuscitation strategy rather than the conventional strategy of aggressive hydration. Nevertheless, the optimal fluid resuscitation strategy in AP remains a matter of debate, requiring further well-designed RCTs, According to some authors, future strategies may be based on the development of individualized resuscitation strategies following a multi-step therapy involving resuscitation, optimization, stabilization, and, in patients exhibiting signs of fluid intolerance and once fluid resuscitation is no longer required, evacuation. The therapy would be tailored to the individual patient’s specific needs, possibly supported by machine learning tools [41].

When dealing with the preferred fluid type, there is generally a stronger agreement. Colloids have not demonstrated a clear superiority to crystalloids, while being associated with more adverse effects, and are therefore generally not suggested [48]. LR has a pH of approximately 6.5, higher than that of NS, approximately 5.5. This pH disparity, together with the distinct electrolyte composition, may contribute to reducing the risk hyperchloremic metabolic acidosis. Furthermore, LR provides sodium lactate as a bioenergetic supplement, potentially mitigating cellular damage [48]. Lactate has also demonstrated anti-inflammatory properties, which may further contribute to reducing the pancreatic damage [9]. Finally, the calcium present in LR has been demonstrated to ionically bind non-esterified fatty acids, which are correlated with the severity of the disease [49]. Based on these physiological considerations and available clinical evidence, both ACG and international guidelines recommend LR over NS for initial fluid resuscitation in AP [9,23]. Several meta-analyses have explored this question. The meta-analysis by Hong et al. reported lower odds of moderate or severe AP, lower rates of ICU admission, and a lower rate of local complications in the LR group. No significant difference was observed in the overall length of hospital stay in the pooled analysis; however, subgroup analysis including only RCTs showed a shorter in-hospital stay in the LR group. No significant differences were found in mortality, organ failure, or SIRS [50]. Likewise, the meta-analysis by Mosquera et al., which included five RCTs conducted across different geographical regions, showed that LR was significantly associated with a reduced probability of admission to the ICU, a reduced risk of progression to more severe forms, and reduced levels of CRP at 48 h. No significant differences were observed in the length of hospital stay or the incidence of SIRS, and the overall quality of evidence was graded as low to moderate, except for the reduction in levels of CRP [51]. In line with previous evidence, the meta-analysis by Zhao et al. demonstrated a significantly lower risk of developing moderate-to-severe or severe AP, lower rates of ICU admission, and a lower incidence of local complications or pancreatic necrosis in patients receiving LR. Sub-group analysis restricted to RCTs also suggested a shorter hospital stay in the LR group. As in the study by Mosquera et al., no significant differences were observed in incidence the of SIRS or in-hospital mortality [52]. Nevertheless, several limitations should be considered when interpreting these findings. In some RCTs, patients with relevant comorbidities, such as pre-existing chronic renal failure or lung and liver disease, were excluded. Moreover, pooled analyses rarely stratified outcomes according to AP severity; furthermore, differences in fluid administration rates were often not accounted for, representing potential confounding factors. In conclusion, the available literature appears to support the administration of LR over NS in patients with AP, although the level of certainty remains moderate. Lastly, it should be noted that in specific clinical scenarios, like in case of hypercalcemia, NS is suggested by international guidelines [23].

Pain management is crucial. Nonsteroidal anti-inflammatory drugs (NSAIDs) are typically as effective as opioids in providing pain relief, with comparable adverse events [53]. Furthermore, a Cochrane review and meta-analysis found no difference in the risk of pancreatic complications and clinical adverse events between opioids and other analgesic options, even if opioids were associated with a lower need for supplementary analgesia [54]. Conversely, a multicenter international prospective study found a cumulative dose-dependent relationship between opioid use and the severity of AP; however, a sub-analysis of daily doses found no differences, suggesting possible confounding factors associated with longer hospital stays of patients with severe AP [55]. The analgesic of choice exhibits significant variations across different countries. In Europe, NSAIDs are employed in approximately 68% of patients, whereas in India, tramadol emerges as the preferred analgesic in around 91% of patients. In North America, opioids constitute the cornerstone of analgesia, being utilized in up to 93% of patients [7]. In accordance with international guidelines, both therapies are valid alternatives. However, in cases of severe pain, opioids are typically recommended, with particular attention given to patients with altered mental status, paralytic ileus, or respiratory depression, in which scenarios opioids should be avoided [23].

Patients with AP are at an elevated risk of malnutrition due to the increased catabolic expenditure associated with the inflammatory state, particularly in severe cases. However, even patients with mild AP must be considered at nutritional risk, especially those in whom pre-existing malnutrition is present, such as those with increased alcohol consumption [56]. Traditionally, it was believed that enteral nutrition in an inflamed pancreas would stimulate its secretion, thereby worsening the disease. According to this principle, parenteral nutrition was extensively utilized in the past [6]. Subsequent evidence has shown that enteral nutrition exerts a trophic effect by preserving gut mucosal integrity and reducing gut bacterial translocation [8]. A Cochrane meta-analysis published in 2010 compared enteral and parenteral nutrition and found a significant reduction in mortality (RR 0.39), a decreased risk of multiple organ failure (RR 0.55), and a reduced incidence of infection (RR 0.39) in patients receiving enteral nutrition. None of the included RCTs were blinded; nevertheless, the results were consistent and contributed to the transition toward enteral strategies [57]. Similar findings have been confirmed in subsequent research. In a network meta-analysis, Hsieh et al. reported lower mortality in patients receiving enteral nutrition via feeding tubes compared with both *nil per os* and total parenteral nutrition, with an additional benefit when nutritional support was initiated within 24 h. Interestingly, in severe AP, enteral feeding was associated with improved outcomes compared with both fasting and parenteral nutrition, whereas no significant mortality reduction was observed in patients with non-severe disease. The large number of RCTs included represents a major strength of this analysis. However, the wide time span covered by the studies causes substantial heterogeneity, particularly in severity classification. Furthermore, information on caloric targets was absent, and blinding was not feasible [58]. By contrast, a large Japanese cohort study reported lower mortality with early enteral nutrition only in patients with biliary AP. However, this association may reflect differences in clinical management rather than a true etiological effect, as patients with biliary disease usually undergo early treatment of gallstones, which may shorten fasting periods [35]. Overall, these findings support early enteral nutrition as the preferred strategy in AP. Current guidelines from the European Society of Clinical Nutrition and Metabolism (ESPEN), together with the ACG and International guidelines, recommend early oral feeding according to patient tolerance. Oral feeding should ideally occur within 24 h, and should not be delayed beyond 72 h when feasible. The initial diet should consist of a low-fat solid diet, as it has been demonstrated that there is no benefit starting with a liquid one [9,23,56]. More recently a normal-fat diet has also been proposed as a possible alternative, as it allows for higher caloric intake without safety concerns, although the available evidence is still limited [59]. Around 26% of patients have been shown to have enteral nutrition intolerance, in particular in those with diabetes, pancreatic necrosis, elevated pre-feeding serum lipase levels, peripancreatic fluid collections and SIRS at admission [60]. When oral nutrition is not tolerated or in cases of insufficient oral intake, nasogastric (NG) or nasojejunal (NJ) feeding tubes are recommended. Continuous administration is generally preferred over bolus or cyclic feeding [9,23]. A recent meta-analysis found no significant difference in mortality rates among patients receiving nutrition via NG or NJ feeding tubes, highlighting how both modalities are effective as nutritional support. Nevertheless, NJ tube positioning has been associated with a lower success rate and increased complications due to the inherent nature of the procedure. Conversely, NJ tubes may be indispensable in cases of vomiting, gastroparesis, or gastric outlet obstruction, where NG tubes may not be feasible [61]. Similar findings were also reported in the network meta-analysis by Hsieh et al. [58].

An exception to early feeding is hypertriglyceridemia-associated AP. In these patients, nutritional support is usually introduced at least 48 h after admission, as fasting alone often reduces triglyceride levels rapidly [23]. Lastly, it should be noted that patients discharged despite persistent nausea after early refeeding may have a higher risk of readmission for recurrent AP [9]. Table 1 reports comparative management strategies of the cited guidelines.

### 4.2. Necrotic Pancreatitis and Pancreatic Collection

As previously mentioned, many patients may develop pancreatic or peripancreatic collections, either fluid or necrotic. Historically, invasive management was more commonly pursued, often involving surgical approaches, which were associated with substantial morbidity and mortality. More recently, however, a more conservative, step-up approach has been adopted, favoring delayed intervention to allow for maturation of the collections [62]. Current guidelines consistently recommend intervention only when collections become symptomatic or fail to improve, such as in the presence of infection, persistent pain, or compression of adjacent structures, and, in selected cases, when progressive enlargement is documented [9,23,63,64]. When intervention is required, minimally invasive techniques, including percutaneous radiologic drainage and endoscopic drainage, are preferred [62]. In particular, endoscopic approaches have gained increasing prominence due to their high efficacy and more favorable safety profile.

**Table 1:** Comparative management.

Domain	AGA 2018 Guideline on Initial Management [44], AGA 2020 Best Practice [63]	ACG 2024 Clinical Guideline [9]	ESGE 2018 [64]	IAP/APA 2025 Updated Guidelines [23]
**Preferred solution**	No recommendation	LR recommended as first-line crystalloid Conditional—low quality	LR as initial fluid Strong—moderate quality	LR as first-line solution Strong—moderate quality
**Infusion strategy**	Goal-directed Conditional—very low quality	Moderately aggressive fluid resuscitation Conditional—low quality	Goal-directed (5–10 mL/kg/h) Strong—moderate quality	Moderate fluid infusion rate of 1.5 mL/kg/h. Bolus if the patient has hypovolemia or hypotension at presentation Strong—moderate quality
**Monitoring targets**	HR, MAP, urine output, Hct, BUN as targets No specific grade	Decrease in BUN and Hct within 6–8 h; reassess every 6 h over first 24–48 h Conditional—low quality	HR <120/min, MAP 65–85 mmHg, urine output >0.5 mL/kg/h, Hct <44%, declining BUN; maintenance of creatinine levels Weak—moderate quality	MAP 65–85 mmHg, urine output 0.5 mL/kg/h, BUN < 20 mg/dL and a hematocrit <44 Good Practice Statement
**Oral feeding**	Early (within 24 h) oral feeding as tolerated. Strong—moderate quality	Within 24–48 h; low-fat solid diet without stepwise liquid advancement Conditional—low quality	EN with polymeric nutrition in all patients with predicted severe AP who cannot tolerate oral feeding after 72 h Strong—high quality	As soon as patient has appetite and no vomiting; regular low-fat solid diet safe Strong—moderate quality
**Diagnosis of infected collection**	Gas in collection, bacteremia, sepsis or clinical deterioration. FNA rarely necessary. Best practice advice	FNA not suggested in patients with suspected infected necrosis Conditional—low quality	Routine percutaneous FNA not recommended Strong—moderate quality FNA only if there is suspicion of infection and clinical/imaging signs are unclear. Weak—low quality	Positive microbiologic cultures, Clinical, imaging (gas bubbles), CRP, white blood cell and procalcitonin Strong—moderate quality
**Intervention timing**	Avoid debridement in first 2 weeks. Delay to 4 weeks optimally; earlier only if organized collection and strong indication Best Practice Advice	Delay ≥4 weeks if clinically stable; antibiotics to delay surgery Key concept	Delay first intervention to 4 weeks if tolerated by patient Weak—low quality	Delayed allowing for encapsulation, around ≥4 weeks from onset Strong—low
**Preferred approach**	Both percutaneous and endoscopic drainage. Endoscopic transmural drainage of WON preferred, as it avoids the risk of fistula Best practice advice	Minimally invasive methods preferred to open surgery for debridement and necrosectomy in stable patients with symptomatic pancreatic necrosis. Key concept	Endoscopic or percutaneous drainage as first interventional method, based on WON location and local expertise Strong—moderate quality	Endoscopic or percutaneous step-up; minimally invasive surgery if needed Strong—high quality
**Endoscopic stent type (LAMS vs. plastic)**	LAMS superior to plastic stents for WON Best practice advice	No indication	Either plastic stents or LAMS for initial drainage; long-term LAMS data still sparse. Weak—moderate quality	Multiple plastic stents or LAMS, LAMS preferred; Strong—high quality
**Direct endoscopic necrosectomy (DEN)**	DEN if no response to drainage alone Best practice advice	If no response to antibiotics in a short time or if the clinical situation deteriorates, necrosectomy/debridement should be performed Few evidence	If no improvement after endoscopic transmural drainage, prefer DEN or minimally invasive surgery over open surgery. CO_2_ mandatory to reduce gas embolism risk Weak—low quality	If persistent fever or signs of sepsis despite antibiotics and adequate drainage. Or patients with infected necrotizing pancreatitis who fail to improve clinically despite adequate antibiotic treatment and cannot undergo drainage either percutaneously or endoscopically Strong—high quality
**ERCP in biliary AP and** **Cholecystectomy (timing and follow-up)**	No urgent ERCP (<24 h) if biliary AP without cholangitis Conditional—low quality Cholecystectomy during index admission for mild biliary AP Strong—moderate quality	medical therapy over early (within the first 72 h) ERCP in acute biliary pancreatitis without cholangitis Conditional—low quality If complicated by cholangitis ERCP within the first 24 h Key concept Cholecystectomy preferred before discharged. Key concept	Urgent ERCP ≤24 h if cholangitis Strong—high quality Within 72 h if persistent biliary obstruction without cholangitis; not recommended if neither present Weak—moderate quality	Early ERCP not recommended in mild or severe AP without cholangitis. Strong—high/moderate quality Urgent ERCP if pancreatitis Strong—high quality Cholecystectomy during same admission for mild biliary AP Strong—moderate quality

Infection is the most frequent complication, occurring in up to one-third of patients with pancreatic/peripancreatic collections, leading to high mortality. It typically occurs 10–14 days after the onset of AP [8,62]. Antibiotic prophylaxis is not recommended, as available evidence has not shown a clear benefit. However, the strength of the recommendation differs across the main guidelines, with international guidelines providing a strong recommendation based on high-quality evidence, whereas ACG guidelines report a conditional recommendation based on low-quality evidence [9,23]. As a consequence, it is imperative that the clinician ascertains any indicators and signs of infection. High fever, clinical deterioration, exacerbation of leukocytosis, or persistent malaise may suggest a potential infection, although they can also be observed in the context of ongoing SIRS associated with AP, making them hard to distinguish [63]. Positive microbiologic cultures from body fluids or the presence of gas bubbles within the pancreatic/peripancreatic necrotic collection on imaging suggest infection and thus the need of antibiotic therapy. Conversely, isolated elevations in white blood cells, procalcitonin, or C-reactive protein should not be used as biomarkers to guide antibiotic initiation. Routine use of fine needle aspiration (FNA) of the content of the collection, under CT or ultrasound guidance, is not recommended, as it may itself increase the risk of infection [23].

Recent studies have demonstrated that antibiotics alone can lead to the resolution of infection and, in some cases, avoid intervention [9]. For this reason, a step-up approach is generally recommended; however, the optimal timing and sequence of interventions remain debated [9,23,63]. The use of a broad-spectrum antibiotic with adequate penetration into the pancreatic parenchyma is advised, such as carbapenems, third-generation cephalosporins, metronidazole, or quinolones [63]. If the patient remains stable, interventional therapies are delayed for at least 4 weeks, allowing for collections’ wall formation [21]. However, it should be noted that in approximately 43% of the patients, the necrotic collection is already encapsulated, resulting in WON at 3 weeks [65].

Several minimally invasive approaches using endoscopy, radiology, and surgery have been proposed [62]. A multicenter randomized trial by Van Brunschot et al. randomized 98 patients into an endoscopic step-up approach, first with endoscopic drainage and then, if necessary, with endoscopic necrosectomy, and a surgical step-up approach, with radiological drainage and later video-assisted necrosectomy. The endoscopic step-up approach did not demonstrate superiority in mortality rates and major complications, although it was associated with a lower incidence of pancreatic fistulas and a shorter length of hospital stay. However, the relatively small sample size and the need for additional percutaneous drainage in approximately one-third of the patients treated with endoscopic drainage limit the generalizability of these findings [66]. Similar results have been demonstrated in a larger meta-analysis by Mohamadnejad et al., where endoscopic drainage was associated with lower mortality and fewer pancreatic fistulas when compared to both open surgery and minimally invasive surgery [67]. Endoscopic drainage has been reported to have higher clinical success, lower mortality, shorter hospital stays, and fewer re-interventions compared to percutaneous drainage in a meta-analysis by Khizar et al. [68]. Overall, percutaneous and endoscopic drainage are generally considered safer alternatives, even if the endoscopic approach is preferred in order to prevent fistula formation [9,23,63]. However, a combined endoscopic and percutaneous approach may be beneficial in cases involving large or septated collections, or when they are not amenable to endoscopic drainage, such as those involving the paracolic gutter [69].

Regarding the endoscopic approach, either plastic stents or lumen-apposing metal stents (LAMSs) are recommend for initial endoscopic transmural drainage by the European Society of Gastrointestinal Endoscopy (ESGE) [64], while international guidelines tend to favor LAMSs over plastic stents in WON due to their higher efficacy [23]. However, for purely fluid collections, the evidence remains more conflicting regarding the stent type associated with the best efficacy and safety profile [70]. A recent retrospective comparative study, with a sample of 51 patients found no significant differences in clinical outcomes or complication rate between LAMSs and double-pigtail stents, although LAMSs provided a significantly shorter procedure time; however, the retrospective design and limited sample size reduce the strength of these conclusions [71]. Notably, after WON drainage, necrosectomy is unnecessary in more than half of the patients [69]. Italian consensus on endoscopic management of pancreatic fluid collections, together with ESGE and AGA 2020 guidelines, suggests that direct necrosectomy should be considered only when drainage alone is insufficient because of persistent clinical symptoms or biochemical signs and thus subsequent sessions should also be scheduled according to the clinical course [63,64,69].

Surgery remains an option when other strategies have failed or are not feasible. However, given the complexity of patients requiring intervention, it continues to be associated with significant morbidity and mortality. Therefore, complex AP management should be reserved for selected cases and performed in high-volume, tertiary referral centers [72].

### 4.3. Target Therapies and New Perspectives

In biliary pancreatitis, early ERCP is only indicated in patients with concomitant acute cholangitis or jaundice [9,23]. Evidence indicates that ERCP performed within 48–72 h of admission in patients with biliary AP and concomitant acute cholangitis can reduce in-hospital mortality compared to delayed ERCP, whereas in patients without acute cholangitis, mortality is not reduced [73,74]. Therefore, selective use of early ERCP rather than routine use should be considered. ERCP could be beneficial in preventing recurrence if cholecystectomy is not feasible during the same hospital admission. However, both guidelines recommend performing cholecystectomy during the initial admission for mild biliary AP to reduce the risk of recurrence [9,23]. Same-admission cholecystectomy has proven safe and effective in preventing recurrence in patients [75] while also being cost-effective for the healthcare system [9]. Conversely, in patients with severe AP, a higher post-operative mortality rate has been observed, and there is no consensus regarding the optimal timing for cholecystectomy [76]; thus, surgical timing should be individualized based on clinical stability and local expertise. It is also noteworthy that cholecystectomy has been demonstrated to be effective in preventing recurrences in idiopathic pancreatitis, suggesting a possible occult biliary etiology in a subset of these patients [77].

In the context of hypertriglyceridemia-induced AP, in addition to fasting, insulin could potentially play a role, particularly in diabetic patients [23]. An RCT evaluated the combination of insulin plus low-molecular-weight heparin, showing no benefit over insulin alone [78]. Plasmapheresis can also be considered [23]. However, evidence is not consistent, as other studies have not demonstrated a clear benefit from plasmapheresis, insulin, or heparin, limiting support for their routine use [79]. Furthermore, during follow-up, of patients with hypertriglyceridemia, it is relevant to assess possible genetic substrates in order to prevent recurrences. This consideration is particularly important for the most common familial chylomicronemia syndrome but should also include less frequent gene variants [10]. To effectively reduce triglyceride levels, traditional therapies such as statins, fibrates, and omega-3 fatty acids are commonly employed. However, numerous trials have been conducted to explore targeted therapies that include APO-C III inhibitors, Angiopoietin-like 3 Inhibitors, and Fibroblast Growth Factor 21 analogs. These drugs primarily utilize antisense oligonucleotides, small molecules that interfere with mRNA and monoclonal antibodies, with some, such as Volanesorsen, also demonstrating efficacy in reducing the incidence of AP [80]. Real-world data of these drugs are still missing; however, the wide range of pharmacological mechanisms explored makes the development of targeted therapies highly promising, although their clinical role remains to be defined.

Autoimmune pancreatitis can spontaneously improve in some patients. Glucocorticoid therapy is recommended for patients with obstructive jaundice, abdominal or back pain and symptomatic extrapancreatic lesions, with a remission rate of 98%. The recommended dose is 0.6 mg/kg/day for 2–4 weeks, followed by gradual tapering. In some patients, long-term maintenance with low-dose glucocorticoids is suggested to prevent relapses [81,82]. However, promising evidence from a recent study conducted in a large European cohort demonstrated that lower doses used for a shorter duration, 0.4 mg/kg/day administered for 2 weeks, were equally effective in achieving remission [83]. In patients who are unresponsive to steroids or have comorbidities that can contraindicate long-term steroid treatment, rituximab is an effective alternative [81].

Numerous targeted therapies have been proposed, but there is no definitive indication in current guidelines [23]. Drugs have been tested to reduce the inflammatory state. In a multicenter, double-blind RCT, Huang et al. showed that COX-2 inhibitors reduced the incidence and duration of severe AP without increasing adverse events [84]. A recent review has identified potential future applications of nanotechnologies and extracellular vesicles in delivering specific drugs to the pancreatic parenchyma. However, these approaches remain largely experimental and are not yet applicable in clinical practice at present [85].

## 5. Conclusions

Acute pancreatitis is a complex and heterogeneous disease. Although supportive care has improved outcomes, important gaps remain in early risk stratification and targeted therapies. The implementation of updated international guidelines is expected to improve the standardization of care. Artificial intelligence may represent a valuable adjunct in the future; however, robust validation is required before widespread adoption.

Finally, AP should not be considered a self-limited acute condition: 20% of patients with severe AP will develop recurrent episodes, with 35% of them progressing to chronic pancreatitis; furthermore, 35% of patients are diagnosed with exocrine pancreatic insufficiency and the prevalence of diabetes is around 23%. Long-term follow-up is essential to identify recurrence, pancreatic insufficiency, and metabolic complications, highlighting the need for a comprehensive and longitudinal approach to patient management.

## Data Availability

No new data were created or analyzed in this study.

## Abbreviations

ACG, American College of Gastroenterology; ALT, Alanine aminotransferase; AP, Acute Pancreatitis; APACHE II, Acute Physiology and Chronic Health Evaluation II; AUC, Area Under the Curve; BISAP, Bedside Index of Severity in Acute Pancreatitis; BMI, Body Mass Index; BUN, Blood Urea Nitrogen; CBD, Common Bile Duct; CECT, Contrast-Enhanced Computed Tomography; CMV, Cytomegalovirus; CRP, C-Reactive Protein; CT, Computed Tomography; CTSI, Computed Tomography Severity Index; EBV, Epstein–Barr Virus; ERCP, Endoscopic Retrograde Cholangiopancreatography; ESGE, European Society of Gastrointestinal Endoscopy; ESPEN, European Society of Clinical Nutrition and Metabolism; EUS, Endoscopic Ultrasound; GLP1, Glucagon-Like Peptide 1; HCT, Hematocrit; HR, Hazard Ratio; ICU, Intensive Care Unit; IL-6, Interleukin 6; IPMN, Intraductal Papillary Mucinous Neoplasm; LAMS, Lumen-Apposing Metal Stent; LR, Lactated Ringer; MRI, Magnetic Resonance Imaging; MRCP, Magnetic Resonance Cholangiopancreatography; mRNA, messenger RNA; NG, Nasogastric; NJ, Nasojejunal; NS, Normal Saline; NSAIDs, Non-Steroidal Anti-Inflammatory Drugs; PRSS1, Protease Serine 1 gene; LR, Lactated Ringer; RR, Risk Ratio; ROS, Reactive Oxygen Species; SIRS, Systemic Inflammatory Response Syndrome; SPINK1, Serine Peptidase Inhibitor Kazal Type 1; US, Ultrasound; WHO, World Health Organization; WON, Walled-Off Necrosis.
